# Artificial intelligence in assisting pathogenic microorganism diagnosis and treatment: a review of infectious skin diseases

**DOI:** 10.3389/fmicb.2024.1467113

**Published:** 2024-10-08

**Authors:** Renjie Han, Xinyun Fan, Shuyan Ren, Xueli Niu

**Affiliations:** ^1^Department of Dermatology, The First Hospital of China Medical University, Shenyang, China; ^2^Key Laboratory of Immunodermatology, Ministry of Education and NHC, National Joint Engineering Research Center for Theranostics of Immunological Skin Diseases, Shenyang, China

**Keywords:** artificial intelligence, pathogenic microorganisms, infectious skin diseases, auxiliary diagnosis, treatment decisions

## Abstract

The skin, the largest organ of the human body, covers the body surface and serves as a crucial barrier for maintaining internal environmental stability. Various microorganisms such as bacteria, fungi, and viruses reside on the skin surface, and densely arranged keratinocytes exhibit inhibitory effects on pathogenic microorganisms. The skin is an essential barrier against pathogenic microbial infections, many of which manifest as skin lesions. Therefore, the rapid diagnosis of related skin lesions is of utmost importance for early treatment and intervention of infectious diseases. With the continuous rapid development of artificial intelligence, significant progress has been made in healthcare, transforming healthcare services, disease diagnosis, and management, including a significant impact in the field of dermatology. In this review, we provide a detailed overview of the application of artificial intelligence in skin and sexually transmitted diseases caused by pathogenic microorganisms, including auxiliary diagnosis, treatment decisions, and analysis and prediction of epidemiological characteristics.

## 1 Introduction

Infectious skin diseases caused by pathogenic microorganisms are diverse, and many clinical manifestations appear similar. Diagnosis often requires assistance from dermatopathology, and is a complex process that necessitates experienced skin pathology specialists. These intricate procedures make the diagnosis of many skin diseases challenging, particularly infectious skin diseases, where prolonged diagnostic processes can lead to treatment delays. Furthermore, there are a relatively limited number of dermatologists, leading to many diseases being diagnosed and treated by non-specialists, resulting in lower diagnostic accuracy and the likelihood of improper or delayed treatment (Liu et al., 2020). Therefore, there is an urgent need to introduce artificial intelligence algorithms to assist physicians in rapid diagnosis and treatment.

Artificial intelligence (AI) simulates human intelligence using computer systems. This is a new technological science that studies and develops theories, methods, technologies, and application systems to simulate, extend, and expand human intelligence. Machine learning is a subset of AI that enables machines to learn tasks automatically by inferring data patterns. Neural networks are flexible mathematical models that employ various algorithms to identify complex relationships in large databases. Neural networks are currently the most popular machine learning technology, particularly with subtypes such as deep learning and convolutional neural networks (CNNs). We input data in the input layer, process it in a hidden multilayer algorithm, and the processed data is displayed in the output layer. Deep learning can be understood as a computational process with very many hidden layers, rather than a simple neural network with only one or a few layers of nodes between the input and output layers. As the computational power grows, the number of hidden layers can even be stacked indefinitely, resulting in a machine with higher sensitivity and specificity (Jartarkar, 2023). Convolutional Neural Networks are a deep learning model with great success in the computer field, inspired by the biological visual system and designed to mimic the processing of human vision. It uses convolutional operations to capture localized features in an image without being affected by their positions. And unlike traditional feature extraction methods, it does not require manual extraction of features (Li Z. et al., 2022). AI can not only process large amounts of data quickly but also access infinite sources of information, with capabilities for perpetual learning and rapid processing (Lillicrap and Morrissey, 2023). With its powerful functions, AI is widely used in the medical field. AI is often used for medical image recognition and interpretation, such as combining with histopathology to identify specific cells in pathological images, and combining with imaging to identify key features in the images to achieve further assisted diagnosis of various diseases, such as cardiovascular diseases, endocrine diseases, and tumors (Hutchinson et al., 2023; Giorgini et al., 2024; Makimoto and Kohro, 2024). AI is also able to analyze multi-parameter data to develop personalized treatment and care plans that can be referenced, such as combining with radiology to assist in the treatment of diseases such as tumors (Alabi et al., 2024; Bo et al., 2024), and can also be used to improve the efficiency of cardiovascular disease care (Jain et al., 2024). In surgery, AI can automate robotic surgeries, provide computer vision, perform pre-operative risk assessment, and post-operative monitoring (Mirshahvalad et al., 2024). AI is used not only for the classification and recognition of skin diseases but also for epidemiological analysis and predictions, as well as for drug and vaccine development (Russo et al., 2020; Paul et al., 2021; Sung and Hopper, 2023). In dermatology, AI is often used for diagnostic recognition of various skin tumors (Brancaccio et al., 2024; Hartmann et al., 2024), as well as for assisted diagnosis, management, and evaluation of various inflammatory and autoimmune diseases (Doolan and Thomas, 2024; Li Pomi et al., 2024). We carefully searched the literature on AI in the field of infectious dermatology to organize and analyze the applications of AI in this field.

## 2 AI-assisted diagnosis of skin diseases related to pathogenic microorganisms

**Figure 1:**
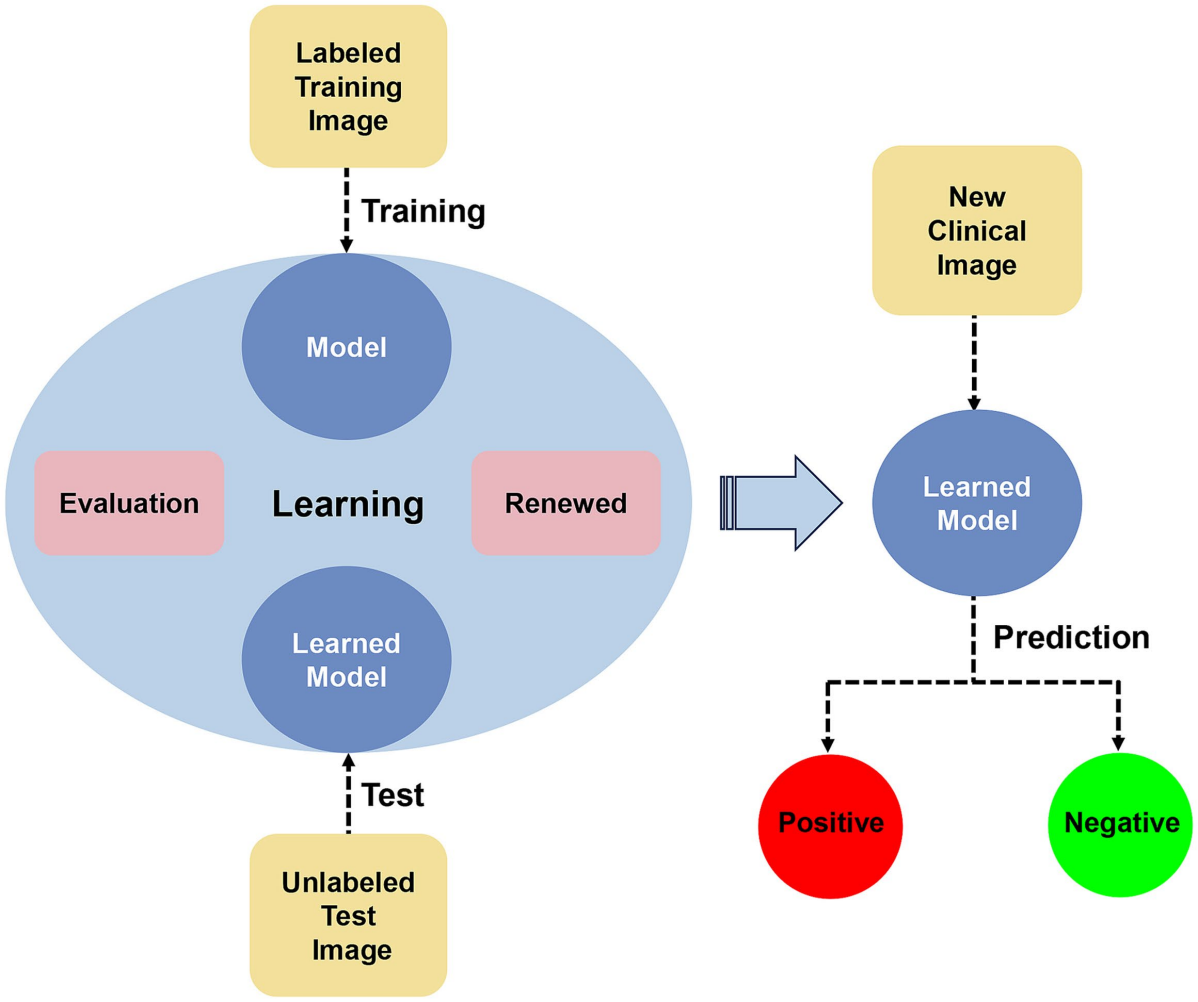
Artificial intelligence-assisted diagnostic model diagram.

The development of AI can assist both professional and nonprofessional individuals in diagnosing and distinguishing diagnoses. By utilizing a large number of clinical case images for training and testing AI algorithms and continuously adjusting and updating them to enhance their sensitivity and specificity, rapid and efficient identification of new case images can be achieved (Figure 1). Currently, there have been some advancements in AI-assisted diagnosis of skin lesions caused by pathogenic microbial infections. We have organized and summarized AI-assisted diagnosis as follows:

Deep CNNs can classify images based on their unique features and are widely used in skin disease classification and diagnostic identification. CNN can be developed and integrated into applications that assist individuals with the diagnosis of monkeypox skin lesions.

Monkeypox, caused by the monkeypox virus (MPXV), is a zoonotic disease (Elsayed et al., 2022). It is characterized by skin lesions that initially present as progressive macules and papules, and later progress to vesicles, pustules, or pseudo-pustules. Thieme et al. (2023) utilized a large dataset of images depicting both monkeypox and non-monkeypox skin lesions to train a CNN algorithm for detecting MPXV skin lesions (MPXV-CNN). The SHapley Additive exPlanations (SHAP) algorithm was employed to identify the regions of high feature importance in the images. In the validation and testing sets, MPXV-CNN exhibited sensitivities of 0.83 and 0.91, and specificities of 0.965 and 0.898, respectively. The effects of factors such as the number of lesions, duration, and site of occurrence on the algorithm have been evaluated (Thieme et al., 2023). An algorithm based on the Al-Biruni Earth radius optimization-based stochastic fractal search was used to fine-tune the CNN, improving its performance from 0.9337 to 0.9883 (Khafaga et al., 2022). Enhanced residual CNNs based on *λ* function and context transformer (LaCTResNet) (Chen and Han, 2023) and Chaos game optimization algorithm-based fusion of deep neural networks (CGO-ensemble) (Asif et al., 2024) were also employed for monkeypox image recognition, enhancing the efficiency of monkeypox identification. McNeil et al. (2023) developed an AI algorithm based on a ubiquitous U-Net deep learning architecture to calculate the number of monkeypox lesions in patient photographs, which employs a segmentation method that categorizes each pixel in each photograph as either belonging to a monkeypox lesion or not belonging to a monkeypox lesion, aiding in monitoring the staging and severity of monkeypox.

Deep CNNs can also be used for the auxiliary diagnosis of skin fungal infections, especially onychomycosis (Han et al., 2018; Kim et al., 2020; Lim et al., 2021; Gupta and Hall, 2022; Zhu et al., 2022; Fang et al., 2023).

Fungal nail disease is caused by fungal infections and leads to discoloration, thickening, and separation of the nail bed (Westerberg and Voyack, 2013). Microscopy and fungal culture are the gold standard techniques for diagnosing onychomycosis; however, they have a relatively high false negative rate (Gupta et al., 2020). The combination of the YOLO v4 deep convolutional network with microscopy enables automation of fungal identification and detection (Koo et al., 2021). The developers trained the target detection convolutional neural network YOLO v4 on microscope images with magnifications of 100×, 40×, and (100 + 40)×. Its sensitivity and specificity were, respectively, 0.952 and 1.0 in the 100× data model, and 0.99 and 0.866 in the 40× data model; the sensitivity and specificity in the combined (100 + 40) × data model were 0.932 and 0.89, respectively, indicating that mycelium was detected with reliable accuracy. Additionally, the integration of the VGG16 and InceptionV3 models with deep CNNs (Yilmaz et al., 2022), as well as image processing models based on residual neural networks (ResNet) (Gao et al., 2021), allows for automatic detection of skin fungi using microscopy. Furthermore, employing AI to assist single-cell Raman spectroscopy technology not only distinguishes between bacterial and fungal skin infections but also identifies fungal species, with researchers reporting a 100% accuracy rate at the strain level (Xu et al., 2023). AI deep learning combined with histopathology can serve as a screening tool to highlight suspicious mycelial areas for rapid confirmation by dermatopathologists (Decroos et al., 2021). Moreover, AI deep learning has been applied in the diagnosis of cryptococcosis (Wei et al., 2023).

For bacterial skin diseases, AI has been widely used in the auxiliary diagnosis of acne (Yang et al., 2021) and leprosy (Barbieri et al., 2022; Fernandes et al., 2023), achieving good results in monitoring, preventing, and guiding patient medication. We made a table to make it easier to see (Table 1). The Inception-v3 network, a deep learning-based classification model, was trained by the researchers using common clinical photographs of acne of varying severity to model the assessment of acne severity and classify the type of lesion based on the image; Inception-v4 and ResNet-50 were also used to train the assessment of leprosy images.

**Table 1:** Application of AI algorithm model in infectious skin diseases.

Pathogen	Disease	AI Algorithm model and features		Application
Virus infection	Monkeypox	CNN + SHAP (Thieme et al., 2023) CNN + BERSFS (Khafaga et al., 2022) CGO-ensemble (Asif et al., 2024) ResNet (Chen and Han, 2023)	Identify the image features; better than clinical CNN	Auxiliary diagnosis
		EPIWATCH system (Hutchinson et al., 2023)	Analyze the epidemiological characteristics	Risk prediction
	Warts	IAPSO for AIRS (Abdar et al., 2019) Fuzzy rule-based system (Khozeimeh et al., 2017)	Predict and evaluate treatment response	Clinical Decision
	AIDS/HIV Infection	GBM, RF, DL, XGBoost (Bao et al., 2021)	Can be used for high-risk populations and individuals	Risk prediction
		ChatGPT 3.5 (Koh et al., 2024)	Provide treatment advice for patients	Clinical Decision
		Logistic regression, RF, AdaBoost (Maskew et al., 2022)	Guide the adjustment of interventions	
		RF, SVM, MLP (Li B. et al., 2022)	Predicted changes in immune function after 9.9 years	Prognosis prediction
		Bayesian Additive Regression Trees (Elder et al., 2021)	Recurrence risk prediction	
		Graph neural network (GNN) (Wang et al., 2023) Simplified Molecular Input Line System (Chavez-Hernandez et al., 2021) Decision trees, Logistic regression, Artificial neural networks (Singh and Su, 2016)	For HIV-1 protease inhibitors	Treatment target prediction
		Artificial neural networks (Conti and Karplus, 2019)	Estimate the breadth of antibodies	Vaccine development
		IDEPI (Hepler et al., 2014)	Predicted antibody epitope	
Fungal infection	Onychomycosis	DCNN+YoLov4 (Koo et al., 2021) DCNN+VGG16 + InceptionV3 (Yilmaz et al., 2022) ResNet (Gao et al., 2021)	Combined with a microscope for automatic detection	Auxiliary diagnosis
	Cryptococcosis	VGG19, MobileNet, InceptionV3, Incept ResNetV2, DenseNet201 (Wei et al., 2023)	–	Auxiliary diagnosis
Bacterial infection	Acne	Inception-v3 (Yang et al., 2021)	–	Auxiliary diagnosis
	Lepra	Inception-v4, ResNet-50 (Barbieri et al., 2022)	–	Auxiliary diagnosis
		RF (Gama et al., 2019)	New case prediction	Risk prediction
		Bayesian networks (de Andrade Rodrigues et al., 2023)	Predict LR probability	Clinical Decision
	Pyemia	Bayesian networks (Komorowski et al., 2018)	–	Clinical Decision

In conclusion, intelligent AI diagnosis can be applied to viral, bacterial, and fungal skin diseases, especially for the rapid identification of monkeypox skin lesions and the differential diagnosis of skin fungi, demonstrating high sensitivity and specificity, and can serve as an important screening tool.

## 3 The application of AI in predicting and monitoring infectious skin diseases and sexually transmitted diseases

Infectious diseases are associated with the presence of pathogenic microorganisms. Understanding their epidemiological characteristics, early prediction, and monitoring of at-risk populations can help in disease prevention and control.

The EPIWATCH system can analyze the epidemiological characteristics of febrile exanthematous diseases such as monkeypox. It uses automated technology to scan large amounts of open-source data from media reports, news releases, official reports, and social media for early warning of emerging infectious diseases (Hutchinson et al., 2023). Additionally, researchers have compared the accuracy of nine AI models in predicting monkeypox outbreaks and provided the details of each model (Chadaga et al., 2023).

Early detection of *Mycobacterium leprae* and its infections is a key factor in breaking the leprosy transmission chain. An AI molecular and serological comprehensive analysis method based on the random forest algorithm can be used to better diagnose and predict new leprosy cases among contacts. Its sensitivity in the diagnosis of polymicrobial leprosy was 90.5%, better than traditional anti-LID-1 (0.632), anti-ND-O-LID (0.579), and especially in oligomicrobial leprosy (70.6%), which also showed a significant increase in sensitivity, with a total specificity of 92.5% (Gama et al., 2019).

AI can also be employed to predict HIV infection risk in high-risk populations, showing significant improvements over traditional prediction methods (Bao et al., 2021). Pre-exposure prophylaxis (PrEP) involves the use of specific antiviral drugs by individuals not infected with HIV before engaging in HIV-susceptible behaviors. Machine learning algorithms have substantial potential to optimize PrEP by enhancing the identification of high-risk HIV populations (Marcus et al., 2020). Risk scores generated by the AI-based risk assessment tool MySTIRisk, in conjunction with the Jördan index, exhibited 86% sensitivity and 65.6% specificity for identifying populations at high risk for HIV/sexually transmitted diseases (Latt et al., 2024). Researchers have utilized the MySTIRisk tool to develop an online self-assessment questionnaire for predicting individual HIV and sexually transmitted infection risks (Xu et al., 2022). Using machine learning, researchers designed an algorithm model based on electronic health records to swiftly identify individuals at higher risk of HIV infection (Burns et al., 2023), ultimately contributing to increased PrEP utilization. AI plays a crucial role in revolutionizing healthcare, demonstrating significant potential for HIV prevention and intervention strategies (Khatami and Gopalappa, 2021; Xiang et al., 2022).

Many studies have utilized new AI algorithms to predict the occurrence of syphilis in high-risk populations, which could potentially serve as tools for controlling and monitoring its spread (Albuquerque et al., 2023).

The application of AI algorithms aids in the early detection of infectious diseases, such as smallpox and leprosy, helping to break the chain of transmission. Predicting high-risk populations for sexually transmitted diseases such as HIV and syphilis is beneficial for the prevention and control of related diseases and for guiding the rational distribution of health resources.

## 4 AI aids in developing better treatment plans for infectious skin diseases and sexually transmitted diseases

In addition to assisting in the diagnosis and monitoring of infectious skin diseases, AI also aids in the development of optimal treatment plans.

These include classifying leprosy cases, ensuring patient compliance with drug therapy, monitoring geographical treatment coverage, and facilitating the early detection of adverse drug reactions and antimicrobial resistance. AI can also help in the early detection of nerve damage in patients with leprosy, thereby aiding disability prevention and rehabilitation planning (Deps et al., 2024). An AI-based leprosy screening cross-platform application can classify cases as paucibacillary leprosy or multibacillary leprosy, assisting professionals in accurate disease classification and determining appropriate treatment methods (De Souza et al., 2021). The leprosy reaction (LR) is a severe inflammatory response in patients with leprosy and is a major cause of permanent nerve damage. Assessing the risk factors for LR in patients is challenging, but AI can be used to predict LR. An AI system developed based on Bayesian networks and utilizing the NETICA software can assess LR risk based on clinical, demographic, and genetic data, thereby effectively guiding clinical decisions. It has an accuracy of 0.827, a sensitivity of 0.793 and a specificity of 0.862 (de Andrade Rodrigues et al., 2023). AI models can also provide personalized and clinically interpretable treatment decisions for sepsis, thereby improving patient outcomes (Komorowski et al., 2018).

Abdar et al. (2019) proposed a novel evolutionary computer-aided diagnosis (CAD) system, whose main architecture is a combination of improved adaptive particle swarm optimization (IAPSO) and an Artificial Immune Recognition System (AIRS). The CAD system can be used to evaluate the response of warts to immunotherapy and cryotherapy. AIRS is a classification algorithm modeled after the human immune system, and IAPSO has improved the treatment response performance of AIRS by improving the algorithm. Other scholars have utilized Fuzzy rule-based system to predict and assess treatment responses to these two therapies for warts by using information gain to identify the factors that characterize the effective treatment, and then the Fuzzy rule-based system to predict the treatment effect, aiding physicians in selecting the optimal treatment method (Khozeimeh et al., 2017; Singh, 2021).

Trained ChatGPT can provide professional and scientific answers to common treatment queries from HIV-infected individuals, offering consultations and advice on antiretroviral therapy to guide patients through the treatment process (Koh et al., 2024). Machine learning algorithms can also predict and identify HIV patients at risk of treatment interruption and unsuppressed viral load, allowing targeted interventions through differentiated care models to improve cost-effectiveness and prognosis before patients deviate from treatment plans (Maskew et al., 2022). Domínguez-Rodríguez et al. (2022) compared seven machine learning algorithms and found accurate predictions of the prognosis of children with perinatally acquired HIV infection (Domínguez-Rodríguez et al., 2022). AI machine learning models can utilize clinical monitoring indicators to predict changes in the immune function of AIDS patients after 9.9 years of antiretroviral therapy, aiding in patient prognosis assessment (Li B. et al., 2022). Machine learning linked to electronic medical records can be used to predict the risk of recurrent infectious diseases and provide valuable insights (Elder et al., 2021).

In summary, AI can integrate and analyze large amounts clinical, demographic, genetic, and epidemiological data to provide personalized clinical diagnosis and treatment decisions for patients with high-risk infectious diseases such as leprosy and AIDS. It has achieved favorable results in clinical indicator monitoring, disease progression prediction, and cost-effectiveness improvement, thereby providing a more comprehensive perspective on the diagnosis and treatment of infectious skin diseases.

## 5 AI assists in drug development and vaccine research

With the development of computer-aided drug design technology, AI has been successfully utilized for rapid innovation in the virtual screening of candidate drugs (Wang et al., 2023). The application of graph neural networks to predict the antibiotic activity and cytotoxicity of 12,076,365 compounds aids in the selection of molecules with antibiotic activity (Wong et al., 2024). Graph neural networks have also been employed to accurately predict potential therapeutic drugs for HIV-1/HBV coinfection, showing potential applications in multi-target drug virtual screening (Wang et al., 2023). The development of a virtual HIV-1 protease inhibitor library based on natural compound fragments using AI can facilitate the discovery of effective HIV-1 protease inhibitors (Chavez-Hernandez et al., 2021). AI algorithms can also be utilized to predict HIV-1 protease cleavage sites, contributing to the development of HIV-1 protease inhibitors (Singh and Su, 2016; Hu et al., 2022).

Artificial neural networks (ANNs) are a powerful tool that can be used to predict multiple resistances to HIV-1 protease and reverse transcriptase inhibitors (Sheik Amamuddy et al., 2017; Tunc et al., 2023). The EuResist engine was used to forecast responses to anti-HIV treatments, effectively assisting virology experts in selecting effective target drugs for patients carrying drug-resistant HIV strains (Zazzi et al., 2012). A combination of chemoinformatics and artificial neural network methods can be employed to predict and score the activity of ligands that bind to the catalytic core domain of the HIV-1 integrase enzyme (Thangsunan et al., 2016). Regularized ANNs have also been employed to simulate the activity of cyclic urea (a type of HIV-1 protease inhibitor) (Fernandez and Caballero, 2006).

The traditional production of vaccines requires several years and involves high costs. By utilizing AI to assist vaccine development and design, significant time and economic costs can be reduced. Machine learning also plays a role in HIV and antibody research, with AI computational methods used to predict applications in antibodies, neutralizing breadth against multiple viral strains, detecting antibody-virus binding sites, enhancing antibody design, and studying antibody-induced immune responses (Danaila et al., 2022). Machine learning and molecular modeling can also estimate the breadth of CD4bs-targeting HIV antibodies, a method that holds promise for use in the design of HIV antibodies (Conti and Karplus, 2019). By leveraging open-source general machine learning algorithms and libraries, Hepler et al. (2014) developed a software package called IDEPI (IDentify EPItopes) for learning genotype-to-phenotype prediction models from sequences with known phenotypes with the aim of rapidly predicting HIV-1 antibody epitopes and other phenotypic characteristics (Hepler et al., 2014).

With its powerful learning ability, AI can process large amounts of data in a short period, greatly enhancing the efficiency of screening and predicting molecules with pharmaceutical activity and drug therapeutic targets. They can also help to predict the binding sites of viruses and antibodies, evaluate the neutralization potency of antibodies, and play important roles in drug development and vaccine design.

## 6 Discussion

**Figure 2:**
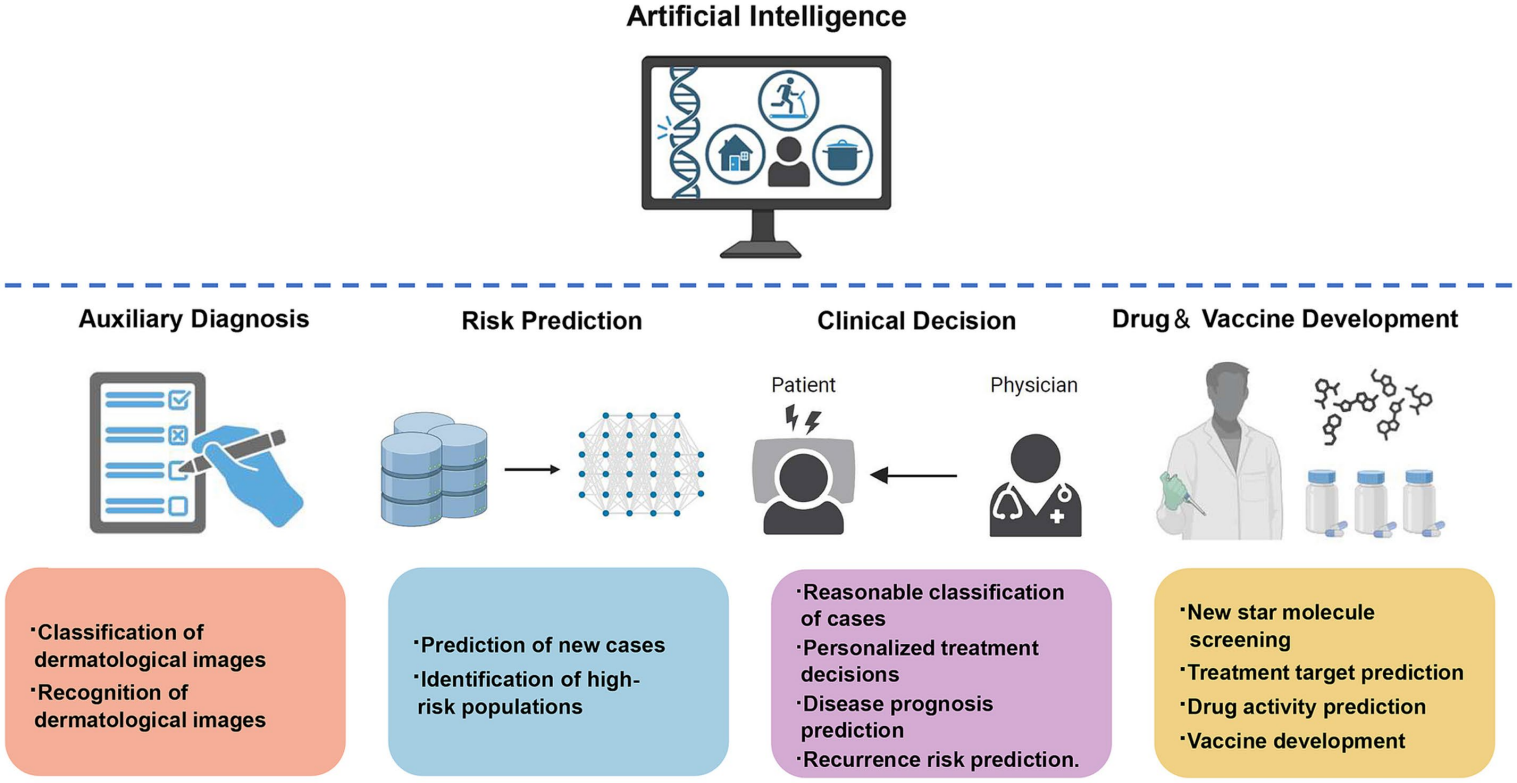
The application of artificial intelligence in infectious skin diseases and sexually transmitted diseases in dermatology.

AI, with its powerful computing and learning capabilities, has become a technological direction with huge potential that profoundly impacts social development and human civilization. AI is widely applied in the fields of infectious skin diseases and sexually transmitted diseases, not only for assisting in diagnosis but also for helping in disease treatment, epidemic prevention and control decision-making, prediction of drug treatment targets, and vaccine development (Figure 2). There are more and more researches on the combination of AI with dermoscopy and dermatopathology in the recognition and diagnosis of infectious skin diseases, however, there are many kinds of skin diseases, and many diseases have similar skin lesion manifestations, which makes it easy for even experienced dermatologists to make mistakes, therefore, AI still needs to be improved in terms of accuracy in the assisted diagnosis of dermatological diseases, and perhaps more dimensional parameters other than pictures can be added to improve the diagnostic efficiency and lead toward more precise diagnosis and treatment. Additionally, the application of AI is mostly in the research stage at present, and the types of skin diseases involved are not comprehensive; the algorithms of AI rely on the selection of reasonable parameters, and their learning mode is limited by the quality of the received information, with various factors affecting whether the algorithm produces an impact, all of which need to be carefully and rigorously tested. In addition, ethical issues in the application of AI need to be pondered, which may involve issues such as data security, privacy invasion, and the lack of standardized regulations (Goldust and Grant-Kels, 2024; Gordon et al., 2024), which may require safeguards to ensure the sound application of AI. Overall, AI is constantly progressing, and these limitations will receive more attention and discussion in the future. It is expected that in the future, AI algorithms and computing power will continue to improve; be applied to more skin diseases; computer science, biology, and medicine more cross-field cooperation, and joint participation in the research and application of AI in dermatology and venereology, improve the effectiveness of disease diagnosis and treatment; effectively reduce the health, psychological, and economic burden on patients; and make greater contributions to human health.
